# Genomic Stability of *Aggregatibacter actinomycetemcomitans* during Persistent Oral Infection in Human

**DOI:** 10.1371/journal.pone.0066472

**Published:** 2013-06-18

**Authors:** Ruoxing Sun, Weerayuth Kittichotirat, Justin Wang, Minnie Jan, Weizhen Chen, Sirkka Asikainen, Roger Bumgarner, Casey Chen

**Affiliations:** 1 Division of Periodontology, Diagnostic Sciences and Dental Hygiene, Herman Ostrow School of Dentistry of the University of Southern California, Los Angeles, California, United States of America; 2 Department of Microbiology, University of Washington, Seattle, Washington, United States of America; 3 Systems Biology and Bioinformatics Research Group, Pilot Plant, Development and Training Institute, King Mongkut's University of Technology Thonburi, Bangkhuntien, Bangkok, Thailand; 4 Department of Oral Microbiology, Umea University, Umea, Sweden; East Carolina University School of Medicine, United States of America

## Abstract

The genome of periodontal pathogen *Aggregatibacter actinomycetemcomitans* exhibits substantial variations in gene content among unrelated strains primarily due to the presence or absence of genomic islands. This study examined the genomic stability of *A. actinomycetemcomitans* during its persistent infection in the same host. Four pairs of *A. actinomycetemcomitans* strains, each pair isolated from an individual over time (0–10 years), were examined for their gains/losses of genes by whole genome sequencing, comparative genomic hybridization by microarray and PCR analysis. Possible effects due to genomic changes were further assessed by comparative transcriptome analysis using microarrays. The results showed that each pair of strains was clonally identical based on phylogenetic analysis of 150 core genes. A novel 24.1-Kb plasmid found in strain S23A was apparently lost in the sibling strain I23C. A 353-bp inversion affecting two essential genes of the serotype-specific gene cluster was found in the serotype antigen-nonexpressing strain I23C, while the same gene cluster was intact in the serotype-expressing sibling strain S23A. A 2,293-bp deletion affecting a gene encoding oxaloacetate decarboxylase and its neighbor region was found in strain SCC2302 but not in the sibling strain AAS4a. However, no evidence of gains or losses of genomic islands was found in the paired strains. Transcriptome profiles showed little or no difference in the paired strains. In conclusion, the genome of *A. actinomycetemcomitans* appears to be relatively stable during short-term infection. Several types of genomic changes were observed in the paired strains of *A. actinomycetemcomitans* recovered from the same subjects, including a mutation in serotype-specific gene cluster that may allow the bacteria to evade host immune response.

## Introduction

Gram-negative *Aggregatibacter actinomycetemcomitans* comprises discrete clonal lineages represented by different serotypes of a to g [1], [2], [3]. We have found substantial genomic variation among unrelated *A. actinomycetemcomitans* strains; the gene content (annotated genes) may differ as much as 19.5% between strains [4]. A majority of the variation are attributed to the presence of specific genomic islands in individual strains. In total, 171 genomic islands (5 Kb or longer) have been identified among 14 *A. actinomycetemcomitans* strains. Cumulatively, 777 strain-specific genes found within these islands represent 61% of the accessory gene pool of the species [4].

The gains and losses of genes, including genomic islands, constitute a major driving force in the evolution of bacteria to enhance their fitness or virulence. The majority of studies of microbial evolution have examined genomic changes occurring over a time scale of thousands to millions of years. These studies typically compared the genomes of strains collected from different geographic regions and biological niches and often with supporting data regarding known times of major ecological events and data from the fossil record [5], [6], [7]. Relatively few studies have examined the microevolution of bacteria. Haubek et al. [8] studied 82 *A. actinomycetemcomitans* strains from different geographic regions by multilocus sequence analysis of six house-keeping genes. The results suggested that strains belonging to a virulent JP2 clone first emerged in North Africa approximately 2,400 years ago, and then spread worldwide through its association with individuals of African descent. Even fewer studies have examined microevolution over the time scale of a persistent infection in the same individual host (tens of years). In this regard, *Helicobacter pylori* is one of the best characterized bacterial species in its microevolution. Kennemann et al. [9] examined four pairs of isolates of *H. pylori* recovered from four chronically infected Colombians (isolation intervals of 3–16 years) by whole-genome sequencing (WGS), and found horizontal gene transfer to be a major factor responsible for genomic changes. Morelli et al. [10] examined 39,300 bp in 78 gene fragments from 34 pairs of *H. pylori* strains from the same individuals (isolation intervals 0.25–10.2 years). Recombination due to horizontal gene transfer accounted for three times as many substitutions as point mutations. Extensive gains and losses of genes were also confirmed by comparative genomic hybridization (CGH) with microarray in *H. pylori* strains isolated from a single individual (isolation interval: 6 years) [11]. Bacterial pathogens may also undergo large scale genome rearrangements or hypermutation *in vivo*, as has been documented for *Pseudomonas aeruginosa* [12], [13], [14]. Lastly, the *in vivo* mutations of bacterial pathogens may be more easily revealed by transcriptome analysis than by WGS or CGH. As an example, Huse et al. [15] examined series of *P. aeruoginosa* isolates from three individuals by transcriptomic profiling and identified 24 genes that appeared to be regulated by the bacterium to enhance its chronic colonization in cystic fibrosis lung. Collectively, the results of these studies suggest that bacterial pathogens are capable of undergoing genome changes during chronic infection as an adaption to the host.

The objective of this study was to examine the gains or losses of genes and genomic islands within strains of *A. actinomycetemcomitans* during persistent infection in the same individual. The changes, if observed, may represent adaptation mechanisms of *A. actinomycetemcomitans* for persistent infections typical of this pathogen. Four pairs of *A. actinomycetemcomitans* strains, each pair isolated from the same individual at time intervals of 0–10 years, were subject to whole genome sequencing, comparative genomic hybridization, and comparative transcriptome analysis. The results showed limited changes of gene content in three pairs of strains and no evidence of changes involving genomic islands were found for any pair of *A. actinomycetemcomitans* strains. Notably, one of the detected genomic changes may allow *A. actinomycetemcomitans* to avoid the host immune response.

## Materials and Methods

### Paired Strains (First Strain/Second Strain) and Genomic DNA Preparation

Four pairs of clinical *A. actinomycetemcomitans* strains (SCC393/A160, SCC1398/SCC4092, SCC2302/AAS4a, S23A/I23C) were included in this study. These strains were part of a historical collection of *A. actinomycetemcomitans* strains by one of the coauthors (SA) and have been used in a series of published studies to examine the clonality, the transmission, and the persistent infection of the species [16], [17], [18], [19], [20], [21], [22]. These studies were approved by the Ethics Committee of the Institute of Odontology, University of Helsinki, Finland. The strains were verified as *A. actinomycetemcomitans* by a 16S rDNA-based PCR assay [23]. Their serotypes were determined by immunodiffusion assay [16], [24]. In three cases (SCC393/A160, SCC1398/SCC4092, SCC2302/AAS4a) the two strains in a pair were isolated from the same subject at two different time points. In the last case (S23A/I23C), the two strains in the pair were isolated from the subject at the same time point, but displayed different serological reactivity to serotyping by immunodiffusion (Table 1). The presence of serotype b specific gene cluster in S23A and I23C was confirmed by PCR [25]. Henceforth, the strains from each individual will be referred to as “paired strains”. Also, the individual strains may be denoted by their sequence of isolation as “first strain/second strain,” or simply as “sibling” strains.

**Table 1:** Four sets of paired strains of *A. actinomycetemcomitans* recovered from four individuals.

First strain	Follow-up strain	Sampling interval (yrs)	Serotype	Age (yrs old) at the initial sampling; gender; race; country of origin	Smoking status	Diagnosis^a^
SCC393	A160	10	E	40; male; Caucasian; Finland	Occasional pipe smoking	CP, severe
SCC1398	SCC4092	3	B	25; female; Caucasian; Finland	Never smoker	LAP
SCC2302	AAS4a	3	C	33; female; Caucasian; Finland	Never smoker	G
S23A, I23C	N/A	0	B, nonserotypeable^b^	48; male; Caucasian; Finland	Never smoker	CP, mild

a CP, chronic periodontitis; LAP, localized aggressive periodontitis; G, chronic gingivitis.

b S23A is serotype b, and I23C is nonserotypeable by immunodiffusion. The serotype b-specific gene cluster was found in the genomes of both strains.

For genomic DNA preparation, *A. actinomycetemcomitans* bacteria were grown on tryptic soy agar plates with 0.6% yeast extract for two days at 37°C in an atmosphere supplemented with 5% CO_2_, and harvested by washing the bacteria off the plates with PBS buffer. The genomic DNA was then isolated using the Qiagen DNAeasy Blood & Tissue Kit (Cat. No. 69504, QIAGEN) according to the manufacturer’s protocol.

### Genomic Comparison by Whole Genome Sequencing (WGS)

The WGS of the four pairs of *A. actinomycetemcomitans* strains resulted in 61–785 large contigs and 10–30X coverage. Detailed information on the WGS of these strains has been published [26], and sequences of individual contigs can be obtained from the Genetable (http://expression.washington.edu/genetable/script/gene_table_viewer), an online tool that we have created to facilitate comparative genomics studies [4]. Genomic islands of the paired strains were identified by the process described previously [4] and are listed in Table S1. The gene content of the paired strains was compared by manually searching the Genetable database containing all annotated genes derived from WGS of the strains.

### Comparative Genomic Hybridization (CGH)

The gene content of the paired strains was analyzed by CGH using a customized pan-genome microarray of *A. actinomycetemcomitans* as described previously [26]. Briefly, the microarray was designed based on the genome sequences of 18 strains of *A. actinomycetemcomitans.* It consisted of 10,934 probes for 2,676 genes, including 1,762 core genes shared by all 18 strains and 914 accessory genes identified in the genomes of one or more, but not all, strains. The genomic DNA was labeled and hybridized to the pan-genome microarray of *A. actinomycetemcomitans* according to the protocol recommended by Agilent Oligonucleotide Array-Based CGH for Genomic DNA (Agilent Technologies, Palo Alto, CA). Data was extracted from the scanner using Agilent Feature Extractor v10.5 software using protocol CGH_105_Dec08, the background signal for each probe subtracted, normalized by the number of “A” nucleotides in the probe sequence, and then log2-transformed. A specific cutoff point was then selected for declaring gene absence or presence (see Table S2 for the histogram of signal distribution and the cutoff point for each set of the CGH data). The comparative genomic hybridization data discussed in this publication have been deposited in NCBI's Gene Expression Omnibus [27] and are accessible through GEO Series accession number GSE42953 (http://www.ncbi.nlm.nih.gov/geo/query/acc.cgi?acc=GSE42953).

### PCR Analysis

The PCR primers (Table S3) were designed using the online primer design tool Primer 3 (v. 0.4.0) and synthesized by IDT (Integrated DNA Technologies). The 25 µl PCR mixture included 50–100 ng of genomic DNA, 2.5 units of Taq DNA polymerase, final concentrations of 0.3 µM of each primer, and 0.2 mM dNTPs in 1× Taq DNA polymerase buffer (New England Biolabs). The PCR amplification was performed with the following thermocycling profile: 2 minutes at 94°C for denaturation followed by 30 cycles of 94°C for 30 seconds, an annealing step at 52–58°C (depending on the GC content of the primers) for 1 minute, and an extension step at 72°C for 1 minute. The cycles were followed by a final extension of 8 minutes at 72°C. An alternative procedure of PCR used LongAmp Taq (New England Biolabs) for amplification. The 25 µl LongAmp Taq PCR mixture included 10 ng genomic DNA, 2.5 unit of LongAmp Taq DNA polymerase, and final concentrations of 0.4 µM of each primer, and 0.3 mM dNTPs, in 1×buffer. The amplification was performed with the following thermocycling profile: 3 minutes at 94°C for denaturation followed by 30 cycles of 94°C for 30 seconds, an annealing step at 52–58°C (depending on sequences of the primers) for 1 minute, an extension step at 65°C for 5–8 minute (depending on the size of the amplicon). The cycles were followed by a final extension of 10 minutes at 65°C. A core gene of *A. actinomycetemcomitans* (p-cluster09322) was used as a positive control for PCR analysis.

The resultant amplicons were analyzed by electrophoresis in a 1% agarose gel. After gel electrophoresis, selected amplicons were purified as needed with QIAquick PCR Purification Kits (Cat. No. 28106, QIAGEN) directly from PCR products or by QIAquick Gel Extraction Purification Kits (Cat. No. 28706, QIAGEN), and submitted for sequencing (Eton Bioscience Inc, San Diego).

### Sequence Determination of Plasmid

The sequence of *A. actinomycetemcomitans* plasmid pS57 (GenBank Access No. NC_014629) was used to identify and construct a scaffold for the contigs of strain S23A. The contig gaps were then closed by PCR. The PCR amplicons for gaps >700 bp in size were sequenced twice from both ends, and additional primers were designed for subsequent sequencing when gaps were >1,400 bp in size. For the amplicons showing ambiguous sequencing results, long amplification PCR was employed to amplify large fragments spanning several gaps.

### Detection of Plasmid by Gel Electrophoresis

Plasmid DNA from strain S23A was extracted with Qiaprep Spin Miniprep kit following the manufacturer’s recommended protocol (Qiagen, Maryland, USC). A portion of the extracted plasmid DNA was digested with *Sma*I at 25°C for 1 hr following the manufacturer's protocol. Both intact and *Sma*I-digested plasmid DNA from strain S23A were then subjected to electrophoresis in 1% agarose gel and visualized after staining with ethidium bromide.

### Sequence Analysis of the Serotype-specific Polysaccharide Antigen (SSP) Cluster of Strains S23A and I23C

The published sequence of the serotype b SSP gene cluster [28] was used to identify and provide a scaffold for contigs of S23A and I23C. The contig gaps were then closed by PCR.

### Phylogenetic Analysis

Phylogenetic analysis of the 18 *A. actinomycetemcomitans* genomes and *Aggregatibacter aphrophilus* NJ8700 was performed using concatenated sequences of 150 core genes (total alignment length is 127,857 bp) (Table S4). Information of the 10 strains not listed in this study and *A. aphrophilus* NJ8700 can be found in previous publications [4], [29]. The 150 core genes were found in all 18 strains and *A. aphrophilus* strain. These genes were found not to have frameshifts and fragmentation that may confound gene detection and annotation. The maximum likelihood method was used to build a cladogram indicating the relatedness among the strains.

### Comparative Transcriptome Analysis

The protocol for transcriptome profiling with the pangenome microarray has been described previously [26]. Briefly, a starter culture was prepared by inoculating the bacteria as a single cell suspension [30] in tryptic soy broth with 0.6% yeast extract and incubated overnight at 37°C in an atmosphere supplemented with 5% CO_2_. The starter culture (OD_650_ of 0.24–0.30) was then diluted with fresh broth to OD_650_ = 0.1 and incubated further for an additional four hours when the bacteria reached the log phase based on the second measurement of OD_650._ Aliquots of the bacterial cultures were used to check for contamination and also to enumerate the colony forming units after plating on agar.

The bacterial RNA was isolated using RiboPure™-Bacteria Kit (Life technology). The resultant RNAs were checked for DNA contamination by PCR using 16S rRNA primers. The RNAs were labeled using MessageAmp™ II-Bacteria kit (Life technology) and then hybridized to the pan-genome microarray following the recommended protocol. Expression data obtained from the Agilent Feature Extraction Software were processed as previously described. The normalized signal values from probes that were targeting the same gene cluster were finally consolidated into a single median value. Genes differentially expressed between the paired strains with a ratio of 2-fold or greater were identified by t-test (*P*<0.05). The expression data discussed in this publication have been deposited in NCBI's Gene Expression Omnibus [27] and are accessible through GEO Series accession number GSE43074 (http://www.ncbi.nlm.nih.gov/geo/query/acc.cgi?acc=GSE43074).

## Results

### Clonal Identity of the Paired Strains

**Figure 1:**
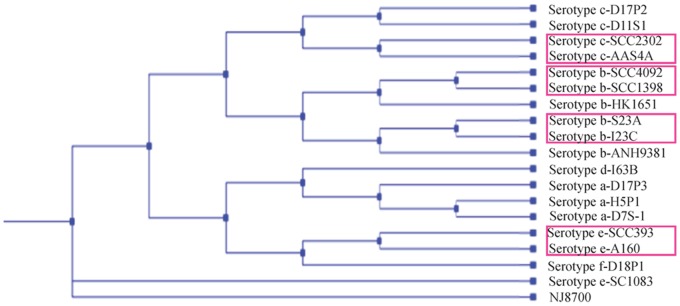
Cladogram of 18 *A. actinomycetemcomitans* strains and *A. aphrophilus* NJ8700 based on 150 core genes. In the cladogram, the paired strains (bracketed by red boxes) were more closely related to each other than to any other strain, indicating that they derived from the same ancestral strain through recent evolution.

It was necessary to rule out the possibility that the paired strains were genetically distinct strains co-infecting the same individuals. Therefore, phylogenetic analysis based on 150 core genes was performed for the paired strains, 10 other sequenced *A. actinomycetemcomitans* strains, and *A. aphrophilus* NJ8700. Three major groups were identified among the *A. actinomycetemcomitans* strains in the dendrogram (Figure 1): (i) serotypes a, d, e (excluding SC1083) and f, (ii) serotypes b and c, and (iii) serotype e SC1083, in agreement with previous findings [4]. Importantly, the paired strains were found in each case to be phylogenetically closer to each other than to any other strain. The clonal identity of the paired strains was further supported by an analysis of single nucleotide polymorphisms (SNPs) within a set of 150 core genes (Table 2). The numbers of SNPs detected between *A. actinomycetemcomitans* strains recovered from different subjects were in the range of 21 to 5,447 (mean ± S.D. of 1,633±1,533). In sharp contrast, no SNPs were detected for pairs of strains from the same individual. The results confirmed that the paired strains were either identical or had derived from a single parental strain in the recent past.

**Table 2:** The numbers of nucleotide differences of the 150 core genes among *A. actinomycetemcomitans* strains and *A. aphrophilus* NJ8700.

	D17P3	D7S-1	H5P1	I63B	SC1083	SCC393	A160	D18P1	HK1651	ANH9381	SCC1398	SCC4092	I23C	S23A	D11S-1	D17P2	SCC2302	AAS4A	NJ8700
D17P3	0	78	80	94	5447	455	455	435	1933	1933	1925	1925	1927	1927	1836	1836	1843	1843	15043
D7S-1	78	0	20	34	5443	436	436	377	1980	1978	1972	1972	1972	1972	1887	1887	1892	1892	15038
H5P1	80	20	0	36	5445	440	440	379	1990	1990	1982	1982	1984	1984	1897	1897	1902	1902	15047
I63B	94	34	36	0	5437	430	430	371	1980	1980	1972	1972	1974	1974	1887	1887	1892	1892	15042
SC1083	5447	5443	5445	5437	0	5421	5421	5414	5398	5377	5388	5388	5371	5371	5387	5387	5393	539­3	15142
SCC393	455	436	440	430	5421	0	0	294	1918	1920	1910	1910	1916	1916	1823	1823	1828	1828	15047
A160	455	436	440	430	5421	0	0	294	1918	1920	1910	1910	1916	1916	1823	1823	1828	1828	15047
D18P1	435	377	379	371	5414	294	294	0	1879	1883	1871	1871	1879	1879	1810	1810	1815	1815	15031
HK1651	1933	1980	1990	1980	5398	1918	1918	1879	0	129	80	80	125	125	718	718	722	722	15068
ANH9381	1933	1978	1990	1980	5377	1920	1920	1883	129	0	121	121	20	20	726	726	730	730	15063
SCC1398	1925	1972	1982	1972	5388	1910	1910	1871	80	121	0	0	117	117	708	708	712	712	15069
SCC4092	1925	1972	1982	1972	5388	1910	1910	1871	80	121	0	0	117	117	708	708	712	712	15069
I23C	1927	1972	1984	1974	5371	1916	1916	1879	125	20	117	117	0	0	722	722	726	726	15060
S23A	1927	1972	1984	1974	5371	1916	1916	1879	125	20	117	117	0	0	722	722	726	726	15060
D11S-1	1836	1887	1897	1887	5387	1823	1823	1810	718	726	708	708	722	722	0	22	42	42	15067
D17P2	1836	1887	1897	1887	5387	1823	1823	1810	718	726	708	708	722	722	22	0	42	42	15069
SCC2302	1843	1892	1902	1892	5393	1828	1828	1815	722	730	712	712	726	726	42	42	0	0	15068
AAS4A	1843	1892	1902	1892	5393	1828	1828	1815	722	730	712	712	726	726	42	42	0	0	15068
NJ8700	15043	15038	15047	15042	15142	15047	15047	15031	15068	15063	15069	15069	15060	15060	15067	15069	15068	15068	0

### Identification of Present/Absent Genes in the Paired Strains

Depending on the sequencing depth and quality, WGS may not have identified all genes. Also, our customized pan-genome microarray did not include all genes found in *A. actinomycetemcomitans* genomes due to difficulty in probe design. Therefore, the concordant results of both CGH and WGS were used to identify 12 genes that were present/absent in the paired strains. An additional 41 genes were selected from among those genes found to be present/absent in the paired strains by WGS that were not included in the probe design for the microarray. These 53 presumptive genes of disparity (defined as genes that were present/absent in the paired strains) and their PCR analysis results are provided in Table S5. Nine of these genes present in S23A but absent in the sibling strain I23C were confirmed (see Figure S1 for the results of PCR analysis). These genes are located on a new plasmid identified in this study, and will be described in more detail in the next section. Ambiguous PCR results were obtained for two presumptive genes of disparity (p-cluster12011 and p-cluster12012) in the paired strains SCC2302/SCC4092 (Table S5). These two genes were carried on a 446-bp contig and encode hypothetical proteins but were not part of a known genomic island of *A. actinomycetemcomitans*. None of the remaining tested genes were found to differ in the paired strains.

### Identification of a Plasmid in *A. actinomycetemcomitans* Strain S23A

The nine genes present in S23A but absent in I23C had significant homology to genes on a known 24-Kb plasmid pS57 of *A. actinomycetemcomitans* strain D11S-1 [31]. Therefore, the sequence of pS57 was used to create a scaffold for 16 contigs in strain S23A. The contig gaps were then closed by PCR primer walk, leading to the identification a 24,102-bp circular plasmid designated as pS23A (GenBank accession no. JX436327) (see Figure S2 for sequencing strategy and the resultant genetic map of the plasmid, and see Table S6 for annotations of the plasmid). The sequence of pS23A was 97% identical to pS57, and 82% identical to another *A. actinomycetemcomitans* plasmid pVT745 in strain VT745. The plasmid was also isolated directly from strain S23A by plasmid DNA extraction and gel electrophoresis (data not shown).

### Presence of a pS23A-homologous Region in *A. actinomycetemcomitans*

During sequencing of the plasmid pS23A, two contigs were found to contain both a pS23A-homologous region and a non-plasmid region. Barring sequencing error, this was interpreted as the existence of a genomic region with homology to the plasmid pS23A. The strategy for confirmation of this plasmid-homologous region in the *A. actinomycetemcomitans* chromosome is provided in Fig S3. The results showed that approximately14 Kb of the 24.1-Kb plasmid pS23A (nucleotide coordinates 11732–24102 and 1–1754) was found in the genome of S23A. A similar approximately 14 Kb pS23A-homologous region was also identified in the genome of strain I23C by the same strategy (data not shown). Moreover, this approximately 14 Kb pS23A-homologous region was found in serotype b strain ANH9381 (>95% sequence homology, nucleotide coordinates 2,125,309–2,136,808 and 1–2,636).

### Identification of Inversion or Deletion of Genes in the Paired Strains

The shared genes in the paired strains may differ in their sequences due to mutations such as insertion, deletion or inversion; such differences may or may not be detected by the approaches described above. Therefore, all present genes in the paired strains were compared by BLAST. Two examples of such mutation were found and described below.

**Figure 2:**
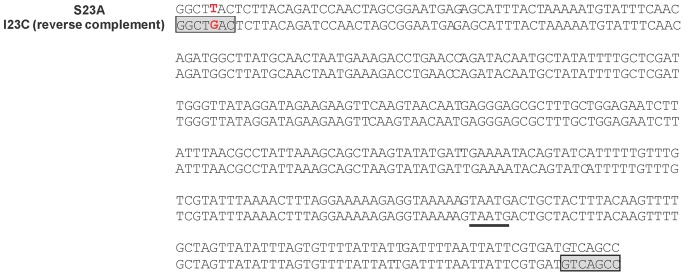
Nucleotide sequences of regions in S23A (upper line) and I23C (lower line) affected by the 353-bp inversion. The stop codon of ORF17 and the overlapping start codon of ORF18 are underlined. The functions of ORF17 and ORF18 have not been defined but both are necessary for the expression of serotype b strain [28]. The displayed sequence of strain S23A is 100% identical to the comparable region in strain Y4 [28]. The single nucleotide mutation in strain I23C is marked red. The resultant 7-base inverted repeats in strain I23C are boxed and shaded.

The SSP gene cluster in strain I23C was found to contain an inversion of 353 bp, while the sequence of the same region was intact in the sibling strain S23A. The relevant regions in S23A and I23C were amplified and sequenced using four sets of PCR primers. The resultant sequences are presented in Figure 2. This 353-bp region in S23A was found to be 100% identical to the published sequence of serotype b strain Y4 [28]. The 353-bp inversion in strain I23C affected the last 278 bases of the ORF17 and the first 76 bases of the ORF18 of SSP gene cluster. The functions of ORF17 and ORF18 are unknown but were found to be essential for the expression of serotype b antigen [28]. The results were consistent with serotype analysis of strain S23A (serotype b) and strain I23C (nonserotypeable) by immunodiffusion assay. Moreover, the 353-bp regions in strain S23A and strain I23C differed by a single base, which converted 5′-GGCTTAC-3′ in S23A to 5′-GGCTGAC-3′ in I23C. Interestingly, this mutation generated a pair of perfect inverted repeats flanking the inverted region in I23C. No such inversion or single-base mutation was found in other serotype b strains in our database.

The BLAST analysis also led to the identification of two homologous genes of different lengths in the paired strains SCC2302 and AAS4a. Sequencing results confirmed a deletion of a 2,293-nucleotide fragment in strain SCC2303 that led to a truncation of 906 bp in the C-terminus of a 1,302-bp gene encoding oxaloacetate decarboxylase, and the deletion of downstream genes of two hypothetical proteins and a bacteriophage Mu GP27-like (see Figure S4).

### Comparative Transcriptomes

Comparative transcriptome analysis may reveal effects due to genomic changes that are difficult to detect by DNA sequence comparison. Therefore, transcriptome profiles of the paired strains were further examined. Strains SCC2303/AAS4a were excluded from transcriptome analysis because of a tendency of SCC2303 to generate smooth-colony variants when cultured in liquid media.

**Figure 3:**
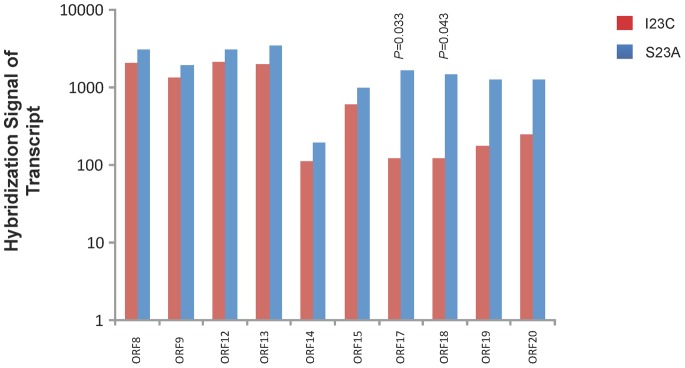
Hybridization signals of transcripts of SSP gene cluster in serotype-b expressing strain S23A and non-expressing strain I23C. Each bar represents mean signal of three biological replicates. Student’s t-test was performed to compare the signals between strains. The low expression levels of ORF17 and ORF18 in I23C could be explained by the 353-bp inversion that affected these two genes in I23C. In contrast, the SSP gene cluster appeared to be intact in S23AS. Note that the two downstream ORF19 and ORF20 also showed decreased levels of expression in I23C but were not statistically significantly different.

Nine, 41, and 87 genes were differentially expressed by t-test (*P*<0.05) for paired strains SCC1398/SCC4092, SCC393/A160 and S23A/I23C, respectively (see Table S7 for the transcriptome data). The numbers of differentially expressed genes were fewer than expected false positives. Significantly expressed genes that reached an expression ratio of two or greater are listed in Table 3. Two genes were found to be differentially expressed in SCC393/A160, with the ratios of their transcripts slightly above two-fold. Seven genes were differentially expressed in strains S23A/I23C, including two genes (ORF17 and ORF18 of the SPA gene cluster) affected by the 353-bp inversion. This 353-bp reversion also resulted in reduced but not statistically significantly different transcript levels of the downstream genes ORF 19–20 (Figure 3). Overall, the results suggested that the transcriptomes of the paired strains exhibited little or no difference.

**Table 3:** Differentially expressed genes of the paired *A. actinomycetemcomitans* strains by transcriptome analysis.

Cluster ID	Expected Length	Product Description	*t*-test; P	Ratio SSC393/A160
**04168**	279	hypothetical protein PM1237	0.0164	0.51
**01144**	1911	ccmF, nrfE : cytochrome c-type biogenesis protein	0.0288	0.47
**Cluster ID**	**Expected Length**	**Product Description**	***t*** **-test; P**	**Ratio S23A/I23C**
**09851**	153	hypothetical protein	0.0013	0.45
**02176**	2421	ATP-dependent OLD family endonuclease	0.0057	0.34
**01731**	1077	CRISPR-associated protein Cas1	0.0067	0.46
**01585**	294	CRISPR -associated protein Cas2	0.02887	0.45
**02262** a	705	ycbB : glycosyl transferase	0.0327	13.77
**02343** a	381	conserved hypothetical protein	0.04282	11.77
**01054**	300	preprotein translocase subunit YajC	0.0470	0.44

a ORF17 and ORF18 were affected by a 353-bp reversion in the serotype specific gene cluster of strain I23C.

## Discussion

This study examined the gains and losses of genes and genomic islands in *A. actinomycetemcomitans* during short-term infection in individual hosts. A comprehensive evaluation of single nucleotide polymorphism in the paired strains we investigated is beyond the scope of this study, and will be pursued in a separate study. To the best of our knowledge, this is the first study that has examined the short-term *in vivo* genomic stability of oral bacteria. The results suggest a relatively stable genome of *A. actinomycetemcomitans in vivo*.

Numerous studies have shown that *A. actinomycetemcomitans* strains isolated from unrelated individuals are genetically distinct [4], [18], [32], [33], [34], [35]. We can reasonably exclude the possibility that the paired strains in this study were unrelated but distinguished by a few minor genetic differences. Therefore, the observed genomic changes in the paired *A. actinomycetemcomitans* strains must have occurred over a short time span *in vivo.* The conclusion can be made without knowing the time interval for the genomic changes to occur, or whether the first strain is the parental strain of the second strain. As an example, a 2,293-bp deletion was found in the first strain SCC2302 but not in the sibling strain AAS4a. It is likely that strain AAS4a was the parental strain (with intact genes), while the SCC2302 was the derived strain with the mutations. However, there is no question that SCC2303 and AAS4a are the same clone.

In this study, *A. actinomycetemcomitans* genome appears to be quite stable during its infection in the same host. There are a number of possible reasons for the differences in the microevolution rates between *A. actinomycetemcomitans* and the well-characterized species such as *H. pylori.* First, *H. pylori* is naturally competent for DNA uptake [36], which could lead to horizontal gene transfer. In contrast, some but not all clonal lineages of *A. actinomycetemcomitans* are naturally competent. A few of the serotypes a, d and e strains but none of the serotypes b or c strains have been shown to be naturally competent [4], [37], [38]. Therefore, the *A. actinomycetemcomitans* strains in this study may be limited in their ability to acquire DNA from other bacteria. Second, genetic exchange between bacteria of the same species may be more efficient than between distantly related bacteria. Infection by multiple strains of *H. pylori* per individual is common in certain populations [39], [40], while co-infection by distinct *A. actinomycetemcomitans* strains is relatively rare [3], [16], [32]. It is likely that the four subjects in this study were each infected by a single *A. actinomycetemcomitans* strain, which precluded the possibility of genetic exchange with other *A. actinomycetemcomitans* strains.

Genomic changes in bacteria may be difficult to detect if they involve small insertions/deletions or minor nucleotide changes of coding and noncoding sequences. However, the effects of such changes may be easily revealed by comparative transcriptome analysis. Silva et al. examined two phenotypically different *Burkholderia multivorans* strains isolated from chronically infected cystic fibrosis patients and identified 392 differentially expressed genes between the mucoid and the non-mucoid strains [41]. Maughan et al. [42] identified differentially expressed genes that regulated sporulation from the transcriptomes of the wild-type and the sporulating-deficient variant of *Bacillus subtilis* after 6,000 generations of evolution *in vitro.* Neither of these studies related the observed differences to specific mutations by DNA sequence analysis. In this study we found relatively few genes that were differentially expressed in the paired strains. The results of the comparative transcriptome analysis may suggest that no other undetected mutations have occurred in the genomes of the paired strains.

A number of studies have reported nonserotypeable clinical isolates of *A. actinomycetemcomitans* [25], [43]. The nonserotypeable strains may be expressing serotype antigens not included in the sets of antisera used in the immunodiffusion assay (e.g., serotype f). Serotypeable strains may also yield nonserotypeable strains while infecting the same individuals [43]. The mechanism of such conversion has not been elucidated. This study showed one possible mechanism for serotype nonexpression due to mutation by inversion that affected the SSP gene cluster. The serotype-specific antigen in *A. actinomycetemcomitans* is an adhesin [44], but may also provide a target for host immune response [45], [46]. It is possible that the mutation in the SSP gene cluster allows *A. actinomycetemcomitans* to avoid the host immune response.

The presence of plasmid-homologous regions in *A. actinomycetemcomitans* has been noted before. Novak and LeBlanc [47] found evidence for the presence of pVT745 plasmid-homologous region(s) in 15 of 35 strains of *A. actinomycetemcomitans* by southern hybridization using the plasmid as the probe. Our study confirmed the previous observations but with more detailed information. The expression and the functions of the plasmid-homologous genes are currently under investigation in our laboratory.

In conclusion, *A. actinomycetemcomitans* demonstrates a greater *in vivo* genomic stability during its short-term persistent infection than other bacterial species such as *H. pylori.* Several types of genomic changes were observed in the paired strains of *A. actinomycetemcomitans* recovered from the same subjects, including a mutation that may allow the bacteria to evade the host immune response.

## Supporting Information

Figure S1 PCR analysis of the candidate genes of differences in the paired strains *A*. *actinomycetemcomitans* S23A/I23C. Genomic DNA from strains S23A (upper) and I23C (lower) were PCR amplified and the products visualized after electrophoresis in 1% agarose and staining with ethidium bromide. Lanes 1–3: PCR products for detection of p-cluster02561 (amplified as three separate gene fragments of S23A_0874, S23A_0875 and S23A_0876). Lanes 4–6: PCR products for p-cluster02280 (S23A_0877), p-cluster02790 (S23A_0936), p-cluster03948 (S23A_0937). Lanes 7–8: PCR products for p-cluster03521 (amplified as two separate gene fragments of S23A_0939 and S23A_0940). Lanes 9–13: PCR products for p-cluster03622 (S23A_0941), p-cluster15527 (S23A_0942), p-cluster02269 (S23A_0872), p-cluster02578, p-cluster02319 (S23A_0938), respectively. Lanes 14–16 were PCR analysis to connect genes located on different contigs. Lane 14: connection between genes S23A_0877 and S23A_0936. Lane 15: connection between S23A_0874 and S23A_0877. Lane 16: connection between genes S23A_0937 and S23A_0939. Lane 17: positive control p-cluster09322.
(PDF)

Figure S2 Identification of plasmid pS23A of strain S23A. The contigs and the regions examined by PCR and primer walk are drawn to scale in Figure S2a. The contigs (large arrows) were scaffolded using the sequence of a plasmid pS57. PCR and primer walk used to close the contig gaps are indicated above the contig by thin lines (regions amplified by PCR) and boxes (sequenced regions). All gaps were sequenced to include at least 100 bp overlapping the ends of the contigs. The final circular genetic map of the plasmid pS23A is illustrated in Figure S2b. It has 42 predicted genes, 38% G+C, and average CDS size of 450 bp.
(PDF)

Figure S3 Strategy for confirmation of a plasmid-homologous region in the genome of strain S23A. The figure is drawn to scale. The arrows depict the direction and location of the contigs. The black lines above the scaffolded contigs represent the regions amplify by PCR. The sequences of the joint regions between the genome and plasmid-homologous regions were determined as needed. The vertical dash lines identify the boundary of the approximately 14 Kb plasmid-homologous regions. Noted that the contigs flanking the plasmid-homologous region contain both plasmid-homologous and non-plasmid sequences.
(PDF)

Figure S4 Sequence comparison of the region of the 2,293 bp deletion in SCC2302 and the intact sequence in the sibling strain AAS4a. The deleted region that occurred in SCC2302 is underlined. The 2,293-bp deletion led to a truncation of 906 bp in the C-terminus of a 1,302-bp gene encoding oxaloacetate decarboxylase, and the deletion of downstream genes of two hypothetical proteins and a bacteriophage Mu GP27-like protein.
(PDF)

Table S1 Genomic islands of the paired strains.
(XLSX)

Table S2 Signals of comparative genomic hybridization by microarray.
(XLSX)

Table S3 Primer sequences.
(DOCX)

Table S4 Cluster ID and product of the 150 core genes used for phylogenetic analysis of *A. actinomycetemcomitans*.
(DOCX)

Table S5 PCR analysis of genes of disparity of the paired strains.
(DOCX)

Table S6 Annotations of the genes found on the plasmid S23A.
(XLSX)

Table S7 Comparative transcriptome analysis of paired strains.
(XLSX)
