# Supplementary figures and images for: Genomic Stability of Aggregatibacter actinomycetemcomitans during Persistent Oral Infection in Human
Source: PLoS One. 2013 Jun 18;8(6):e66472. doi: 10.1371/journal.pone.0066472 (PMC3688926; doi:10.1371/journal.pone.0066472)

1 2 3 4 5 6 7 8 9 10 11 12 13 14 15 16 17

DNA  
Marker

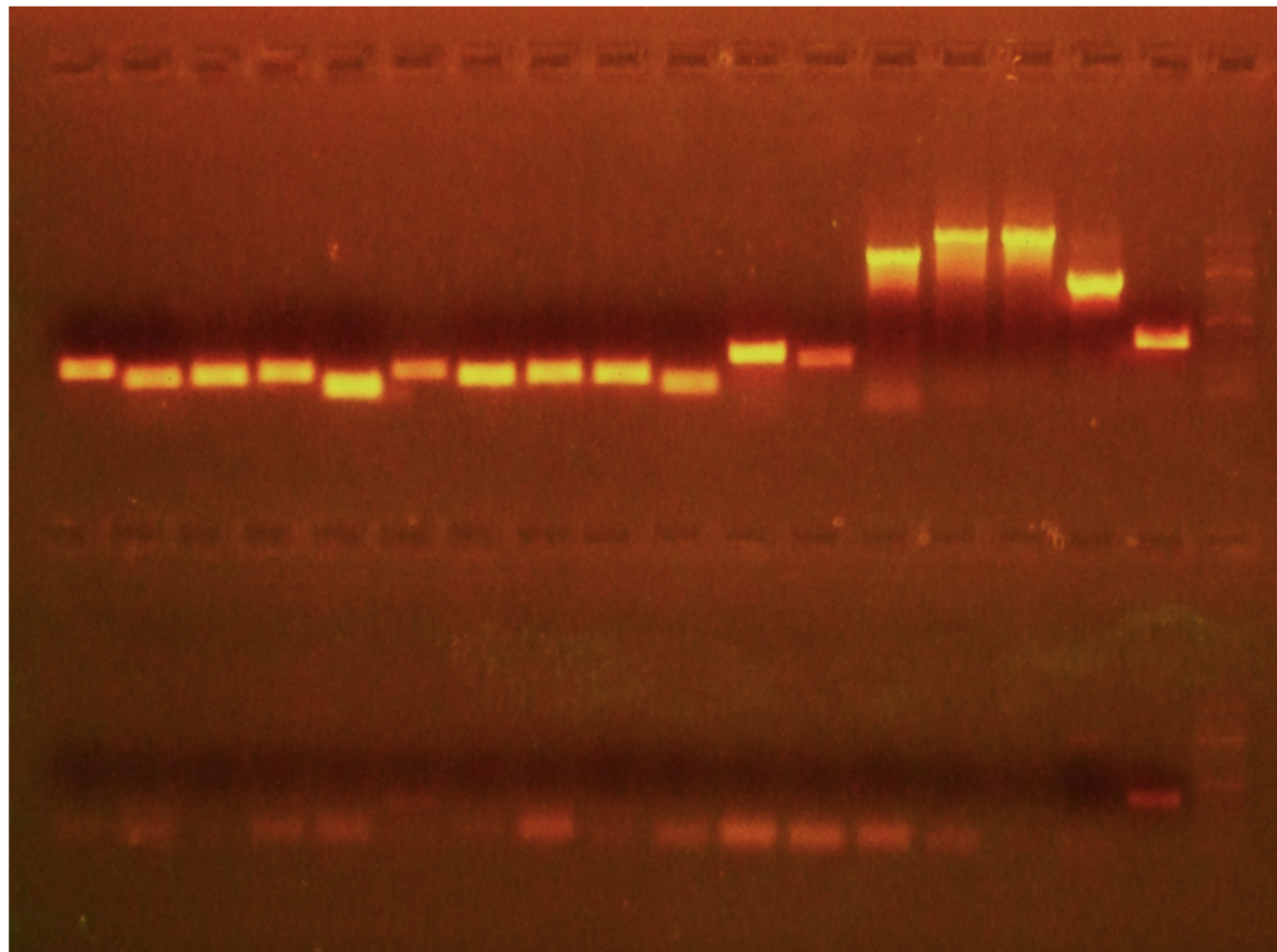

**S23A**

**I23C**

Supplement: Figure S1 — PCR analysis of the candidate genes of differences in the paired strains A. actinomycetemcomitans S23A/I23C. Genomic DNA from strains S23A (upper) and I23C (lower) were PCR amplified and the products visualized after electrophoresis in 1% agarose and staining with ethidium bromide. Lanes 1–3: PCR products for detection of p-cluster02561 (amplified as three separate gene fragments of S23A_0874, S23A_0875 and S23A_0876). Lanes 4–6: PCR products for p-cluster02280 (S23A_0877), p-cluster02790 (S23A_0936), p-cluster03948 (S23A_0937). Lanes 7–8: PCR products for p-cluster03521 (amplified as two separate gene fragments of S23A_0939 and S23A_0940). Lanes 9–13: PCR products for p-cluster03622 (S23A_0941), p-cluster15527 (S23A_0942), p-cluster02269 (S23A_0872), p-cluster02578, p-cluster02319 (S23A_0938), respectively. Lanes 14–16 were PCR analysis to connect genes located on different contigs. Lane 14: connection between genes S23A_0877 and S23A_0936. Lane 15: connection between S23A_0874 and S23A_0877. Lane 16: connection between genes S23A_0937 and S23A_0939. Lane 17: positive control p-cluster09322. (PDF) [file pone.0066472.s001.pdf]

a.

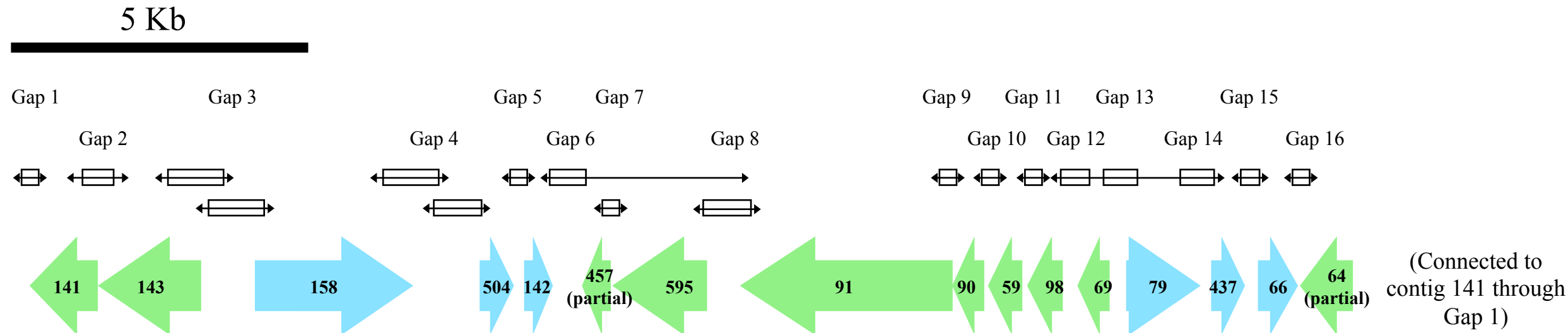

b.

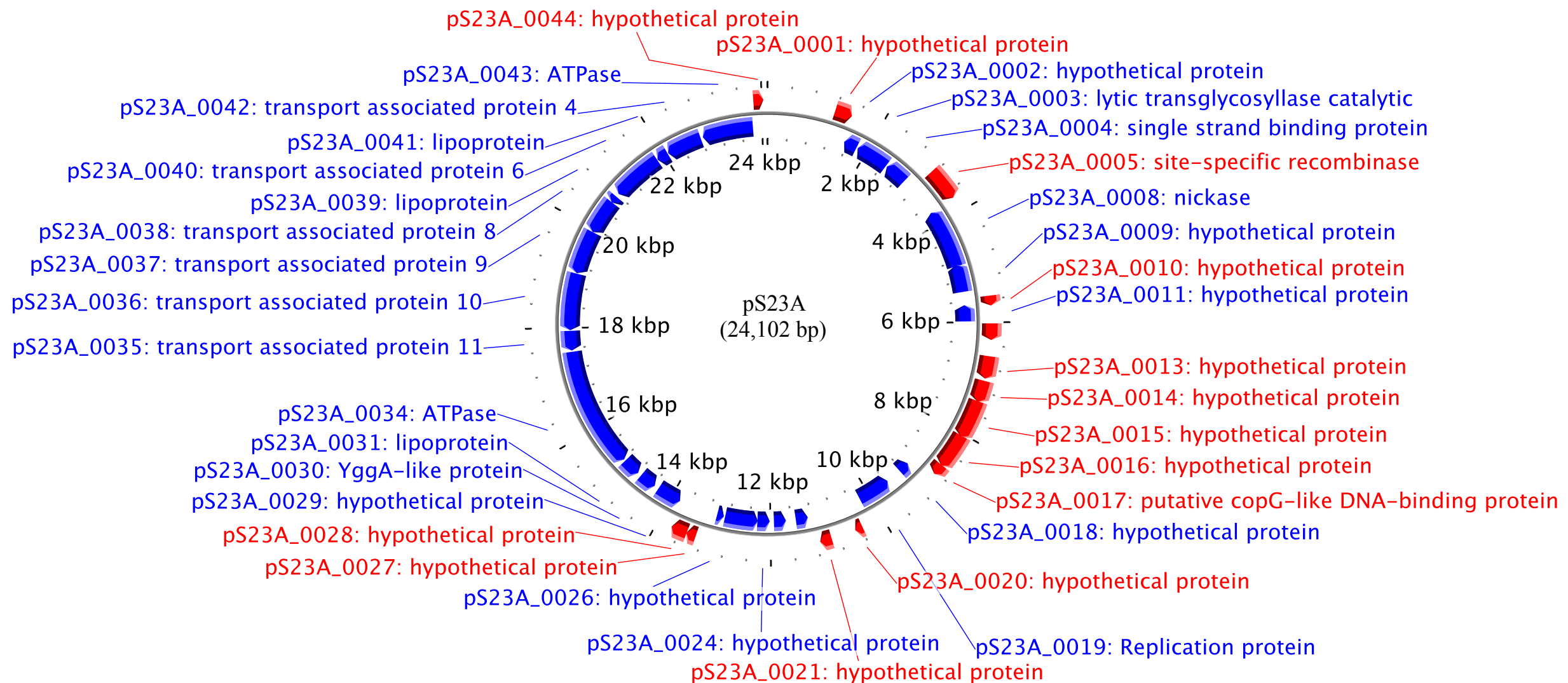

Supplement: Figure S2 — Identification of plasmid pS23A of strain S23A. The contigs and the regions examined by PCR and primer walk are drawn to scale in Figure S2a. The contigs (large arrows) were scaffolded using the sequence of a plasmid pS57. PCR and primer walk used to close the contig gaps are indicated above the contig by thin lines (regions amplified by PCR) and boxes (sequenced regions). All gaps were sequenced to include at least 100 bp overlapping the ends of the contigs. The final circular genetic map of the plasmid pS23A is illustrated in Figure S2b. It has 42 predicted genes, 38% G+C, and average CDS size of 450 bp. (PDF) [file pone.0066472.s002.pdf]

**StrainS23A**

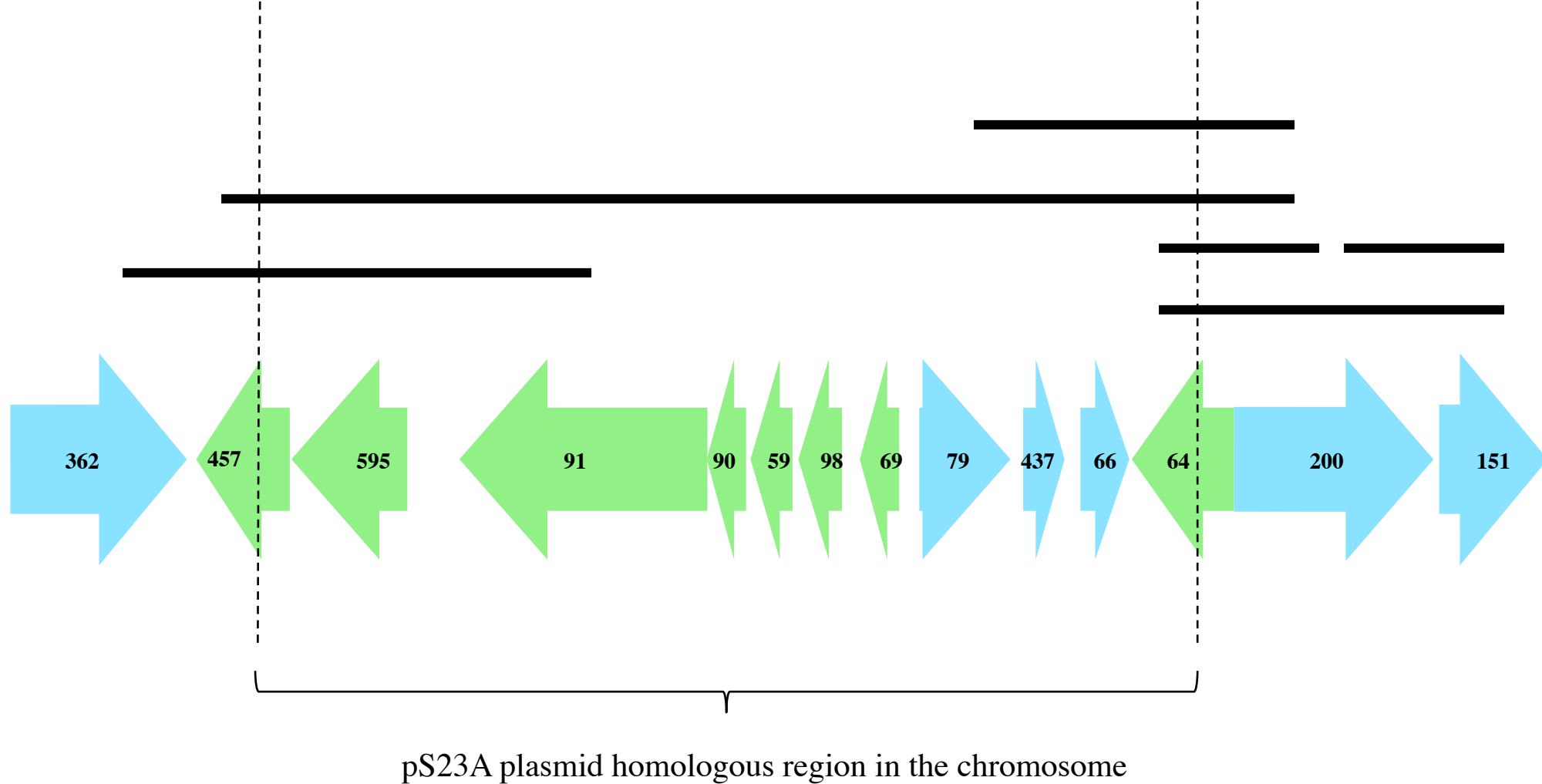

Supplement: Figure S3 — Strategy for confirmation of a plasmid-homologous region in the genome of strain S23A. The figure is drawn to scale. The arrows depict the direction and location of the contigs. The black lines above the scaffolded contigs represent the regions amplify by PCR. The sequences of the joint regions between the genome and plasmid-homologous regions were determined as needed. The vertical dash lines identify the boundary of the approximately 14 Kb plasmid-homologous regions. Noted that the contigs flanking the plasmid-homologous region contain both plasmid-homologous and non-plasmid sequences. (PDF) [file pone.0066472.s003.pdf]
