# Supplementary material for: Genomic Stability of Aggregatibacter actinomycetemcomitans during Persistent Oral Infection in Human
Source: PLoS One. 2013 Jun 18;8(6):e66472. doi: 10.1371/journal.pone.0066472 (PMC3688926; doi:10.1371/journal.pone.0066472)
Supplement: Figure S4 — Sequence comparison of the region of the 2,293 bp deletion in SCC2302 and the intact sequence in the sibling strain AAS4a. The deleted region that occurred in SCC2302 is underlined. The 2,293-bp deletion led to a truncation of 906 bp in the C-terminus of a 1,302-bp gene encoding oxaloacetate decarboxylase, and the deletion of downstream genes of two hypothetical proteins and a bacteriophage Mu GP27-like protein. (PDF) [file pone.0066472.s004.pdf]

TTTAGGCATGTCTAAAAGCGCATTTGCAGAAAGAAAGCGTCGAGGAGTATTTCCTAAAGAAGCATTGATGCTGGCGGATTTAAAACATTCAGAACTAAAAC
TAGACGTCGAATACATTCTAACTGGCAGAAAAGGGGATGAACTGAGTCAACTCTTAACTAGCGTATCTAAAAAGAGGATTGATCACCCTTTAG**ACCCTGAA**
GATATATCAATAGAGGAACAACCAATCACCCCCAAAGATGCGGATGCATACGTTTTAGTGAATATGGAGGAAAAAATTTTATTAAACTGGTATAGAAGAAG
TGATACAAAAGGCAAGAAGCTAATTTTAGATATAGCTAAGATGTCTCACGAAATGCTATTGACACAAATGAGATTGATTGAGTACACAGAAAACAAACTAT
TCAACAACACAGAAAGAGAAAAGCGATAAACTAATAAACCCCCTTTATTTCAAATAGTTACATTAATTACAACTACACAGAAAAAAAACGGAAAACTACAC
AGAATAAAAAATTTACTGTGTACTATATTTAGCACCAACTGGGCGCAGCATTAGCCTTTAATAGATTCATTAAAGAAAGATATTAAAAGCAATAGAAAGAA
CACCTCTCAATTTAGTTAAGCCAATATAAGACAGGAAACCTGCAAATCATCAGGCAAGTCGTTCACATCGGGAAAAACCTTATTTAAAGCGGCTTTAAACG
ATTTTTAAACGCTTTTTAAAGCCTTTCTTTAAATTTCTTAAATCAAGTGGTTAAATTGCACTTTTTAAATTTAGCCGCTTTTTTTCTAATTTTGGTTTTTC
GGTTTTAGGCAAATAAAAAAGGGGCGCCTGCCCCTTTCCTATTTTCCCGTTAAATTCCGAATTATCTCTATAATTTCTTGCCGCCTAAACTCAATTTCTCC
GAGTTCTTTATAAAGCAGATCAATATTGTTATCTTTTGCCGTTAAGCCTGTCATAAGCCTAAAAGACCCCGCCTTTCCATCAAATCCATCTCTTGCTTTTA
ATTGGTTGATGTACCGATTGAGCGCTGATTTAGAGCCTTTTAAGCTGCGTTCTGTTAACCATTTCAAGACTTCATCTTGATTACCGTATTCATTAGTTCTG
ATAACATCATTAAGTTCCAAAAGGACATCCCTTGGCAAAGATGAAATCAAAGATTTTCGACCCATTTTTTCACTCCTTTTAATTTCTTTATTTCCCATTAT
ATTCCTTTATTTATTTTATTCAAGGTCCGCCAATTACAACATAATTCAACAAAATCCCTAGCTTCTTATTTTTCTAATGTTATGTGGTTGGGTACATTAAC
CAATTCAATTTTCTAAACGCGACCCTTTCAATTCGTTCAAATTCTCTTAACTTATTGCTAACCAACATCAATCCTTCGCTTACGGCATAAGCACCAACTTTT
GGTTTGTATGTCGGGTAAAAGAAGAATTAAAGCATACCGGTGGGAAGTAAAAGTGCGGTGGGTTTTTAAATAGATTCTCGCGAGCAAAAAAAAGGCACACG
CCGGTGAAGCGTGAGCCATCATTCTATTTAAAAAGTGCGGTCGGTTTTCGCAACGTTTTTACCGTTACAACATTGCGCCCACATATTTCAGCATCACCCCG
GCGGCAATGGCGGAGCCGATAACACCGGCAACATTCGGACCCATGGCGTGCATGAGCAGGAAGTTTTGGTTATCCGCTTCCAATCCCACCTTGTTGGAGAC
GCGCGCTGCCATTGGCACGGCGGACACTCCGGCGGAGCCGATAAGCGGATTAATTTTGGTTTTGCTGAATTTGTTCATTGTTTTCGCCATTAACACGCCGC
TGCCCGTACCAATGCAGAAAGCAACCACGCCGAGAATTAAAATGCCCAGGGTTTGCGGTTGCAGGAATTTATCGGCAATGAGTTTGGAACCCACGGACAAG
CCAAGCACGATGGTAACGATGTTAATTAACGCGTTTTGGGTGGTGTCGCTTAAACGTTCTACCACGCCGCTCACGCGCATTAGATTGCCGAAACAGAACAT
ACCGAGCAATGGAGCGGCATCTGGCAATAATAAGCCAACAAGCAACAGCAAAATCACCGGAAACAGGATTTTTTCCCGATTGCTTACATGGCGTAATTGGG
TCATGCGGATTTTACGTTCTTCTTCCGTGGTGAGGGCTTTCATTATCGGCGGTTGGATCAACGGCACTAACGCCATGTAAGAATACGCCGCGACGGCAATG
GCGCCGAGTAATTCCGGTGCAAGTTTACTGGTGAGGTAAATTGCCGTCGGACCGTCCGCCCCGCCGATAATGCCGATGGAAGCGGCTTGCGGCAGCGTAAA
TTCAATAATGCCGAAATAATTTAATCCTAACGCGCCAAGCACGGTGGCGAAAATACCGAACTGTGCCGCCGCACCGAGGAGTAGGGTTTTCGGATTTGCCA
GTAACGGACCAAAATCCGTCATGGCGCCCACGCCCATGAAAATCACCAGCGGCGCAATGCCGTAGCCGATAGCGACTTTATAGAACAACGCCAGAATGCCT
GCGCTGTAGCCCATATCCACCGACAAGGTTTCCAACTGGTTAATCATGGAAGTCGGCGCAGAAGCAATGGCTGTTTTGATGATCGCCGGATCAGGCGCAAC
GCCCAATTGCGCGGCAATCACGGCGATTTGTTCCGGCGAGCCAAGGTGGAGCAGGTTTTCCAATGCGCTCATGGCAAGCCCGGCTTCCGGAATGTTTGACA
GCAAACCGCCGAAACCGATAGGCAAAAGCAGCAACGGCTCAAACTTGCGGGCAATGGCAAGCCACAGCAGGAGCAGGCTGATACCAATCATTACCGCCTGC
CCCCATTGCAGGTGAAATATCCCCATGCCTTGAATTAATGCAATAATGCTATCCATAGGGAACTCCT
