# Supplementary material for: Genomic Stability of Aggregatibacter actinomycetemcomitans during Persistent Oral Infection in Human
Source: PLoS One. 2013 Jun 18;8(6):e66472. doi: 10.1371/journal.pone.0066472 (PMC3688926; doi:10.1371/journal.pone.0066472)
Supplement: Table S3 — Primer sequences. (DOCX) [file pone.0066472.s007.docx]

**Supporting Information**

**Table S3.** Primer sequences

**For PCR and sequencing confirmation of genes in SCC393/A160**

| **Cluster ID** | Forward Primer | Reverse Primer |
| --- | --- | --- |
| 13483 | CAGCGCGGTGATCTTCAG | CGATCCTCACCGCGAAAC |
| 02391 | ACAAAAGAAGAGGTGACGGTTGCC | AACCTCGGGCGGGTACTCATT |
| 01053 | GCGCTTCCAAGAAATCACAT | GTTAAAACCGGCAACAAAGG |
| 01138 | CGGGTTAAGGTCGCTCATT | CGGCAACTTGATTAATGCGTA |
| 01300 | GAAACTTATTTGCCCGCTTG | TTGGCATACGTCGGGATTAC |
| 01480 | GGCGGAAcaaCAACAAGATT | CAACCGCATGGTACAACAAG |
| 04441 | AAAGGTAAAAaGTGCGGTGATT | TTATTTGGCAACCGTCACCT |
| 01506 | TTCAGACATACTGCCGCAAC | CATTCTTTGCCATGCTATTTGA |
| 01753 | CCAGAATAAGCCGGTAACCA | AGCAAAAGAAAGCACCTCCA |
| 02140 | TACGAATAGCTTGCGGATTG | GACTTAGGGCCTGCGAAGAT |
| 02923 | GACAACCGCACCACTATCAG | ATTCAGAAACGGACGCAATC |
| 01552 | TGCGGTGGTTTTCTAAAGTG | GAAATCGGCGATGAATCAAT |
| 01556 | AACTGCGTATTGGGACTTGG | AGCCAAAATTTTCGTTGCAC |
| 01651 and 13977 | AGAAACTAGCCATCGCCAAA | CTACCTTTTGGGGCAGATCA |
| 01751 | TCTGCAATTGATTTAGTATTTTtCTGA | GCCCGAGAAGGTGTAATTTTT |
| 01876 | GATATTGTGGGCGGTAATGC | TTGGAATTCTTTTGCTTTTTGA |
| 01894 | GCCACGTACCAGCGTAATTT | AACCGGAGACGGAAGAGAAT |
| 01967 | AACCGGAGACGGAAGAGAAT | CCTTTTTAATGGCGTCCAAA |
| 02004 | CGTGGAGCTCATGAAGCAAT | TTGATGATTTTGGGGGTTATTC |
| 02062 | GCTCCCGTCCAATTTCATTA | CATCGAAAGCAAACTCAGCA |
| 02030 | TCCGCTGTTTTCTTCTAACGA | AAATGCTCCAAACAGCCAAC |
| 02061 | CTGGTGCGCAAATTAAAGGT | GTGAATGCCACGACGACTTA |
| 02110 | TAATCAGCACCGCCTCTTTC | AGGCGTATTGCGtTTTATGG |
| 02134 | CGCCGTTAAGCGTAAAACTT | GGATTCCGCCAATAACCAG |
| 02455 | CGGTCTTCGTCCGTAAAAaC | TCAATACCCGCAATTTTCTT |
| 02841 | AAAGTGCGGTTGATTTTCAC | TAATTCCGGTAGCGGTTCAC |
| 04804 | CGCTTGAGTCAACCAAAGGT | CGGTTTGGTGCATATTCCTT |
| 12497 | TGTTTTTCCCTTTTGCCAAG | AGGTGGCGTTGTTAATTTGC |
| 12703 | GGCGGTAACGTTTCGATTTA | AGGTGCTTTGCCACAATTTC |
| 12864 | GTAGAATGGCGTCGGTGATT | CGCTATGACAAAGCCACTGA |
| 13482, 13483 | GTCGCCGATCGTCTTCAC | GATGCTGTCgAACCAGACCT |
| 13511 | ACAAGGGTGGCAAAGAAGG | CGGGTTTTCATGATCTGCTT |
| 14115 | AGTTAATACCTGCCCCGACA | GACGCAGACTGATTTGGTGA |
| 01065, 02142 | GAGCGAAGCGTCTCGATATT | CCAGATgGGACAGGCAAG |
| 15256, 15257 | ACCCGACAACGAACTGTTTC | ATGTCGCAGATCCTCACGTC |

**For PCR and sequencing confirmation of genes in SCC1398/SCC4092**

| **Cluster ID** | Forward Primer | Reverse Primer |
| --- | --- | --- |
| 01550 | GCCTTCTAGCGTGCCTTTTA | ATTTCACGACGCGCTAAGTC |
| 00457 | CAACCGCAAGAATTTTCAGA | AGGCTGCCTTTGGATTCTTT |
| 01799 | CAAATCAAAAAGGGCTTTCA | TTTTCCCTTTCCTTGTCCTTC |

**For PCR and sequencing confirmation of genes in SCC2302/AAS4a**

| **Cluster ID** | Forward Primer | Reverse Primer |
| --- | --- | --- |
| 12007 | ACCTAAAACGACTGGCAAGC | GAACCGGCtTGAAttTTTGA |
| 12011 | GTGCTTGCTGAATCTATACCAGA | ATAGAGGAGCGCCCCAAC |
| 12012 | TTGAATGTCTCATCTACTGCTCCT | GCGCCTAAAAATGGTTGAAG |
| 03035 | TGCCAAGGGATGTCCTTTTGGAAC | GACCCCGCCTTTCCATCAAATCCA |

**For PCR and sequencing confirmation of the 2,293 bp deletion in strain SCC2303**

| Forward Primer | Reverse Primer |
| --- | --- |
| ATGAACTTTCTGAATTGCAAGG | CAACAGAAGGACAAACCG |
| AGGCAAGTCGTTCACATCG | TCCATCGGCATTATCGG |
| ATCAATCCTTCGCTTACGG | TAACGGTAAAAACGTTGCG |
| TGCGGTGTGTTTTGTAAAAG | AAACGACGGATCACCATC |
| AAGTTGCCCGAGTAAAGCG | ACACCACCAAGAGTGCG |

**For PCR and sequencing confirmation of genes in S23A/I23C**

| **Cluster ID** | Forward Primer | Reverse Primer |
| --- | --- | --- |
| 02319 | GTGGTTGCAAACAAAGCGTA | TCCTCTCCACGCTCCTCTA |
| 02459 | ATAGATGGGCGTTCGTATCG | AAGGTATCTTGCAAAATGTGTTC |
| 01320 | GAAAGGCTCATTGTGGAGGA | GATTTAACAGAACGGCGAAA |
| 01668 | TTTTCTGGTCGGACAACTCC | AACCCATGGCACAAACCTAC |
| 01699 | GTATCCGCACCGTTTGCTAT | TCGCCGTTTTCACGATAGTT |
| 01967 | GGTATATTCGGTGCGCAGAT | CCTTTTTAATGGCGTCCAAA |
| 03035 | TGCCAAGGGATGTCCTTTTGGAAC | GACCCCGCCTTTCCATCAAATCCA |
| 02269 | ATGTGCGCCCCGCTGGTTCA | TCGTTGACACTGCCGCTACGTT |
| 02280 | TTATGCCCGTGTATCGACAA | AGAAACCGGCTTGATTGAGA |
| 02561 | TCATGCTTCATCAAAACCAAA | CGATATTCGCGTTTAACCTCA |
|  | TGGCGATACGGATAATCCTC | TTTCAACAGCTCGGCTAGGT |
|  | GAATATCAACGTGCGAGACG | CTTGTGCAGACGGCAATTTA |
| 02578 | AACGTGGTTTTTCCTGCAAC | AACGCACTCGATTTTTACCG |
| 02790 | AAAATCGAAAAGTTAAGAGAGCAG | AGGATTACTTTCGCATTGTTCC |
| 03521 | CGTAGTGCGCATTTTGGATA | AACAAGACCATGCCCACAGT |
|  | ATCAAGCCACACGGAAAGTT | TCAACTGCTCACGCTTTTTG |
| 03622 | ATGCCGGTCAGAAAACACTC | AGCTGCCGTTAAATCGGATA |
| 03948 | CAAGGCGAATTTTGAGTGGT | TCGCCAAACTCATCATCAAG |
| 15527 | AGCTAAGCAGCACCCGAAA | ATTTCCGCCCGTGATAATTT |
| 02319 | CAAGGCGAATTTTGAGTGGT | CGTAGTGCGCATTTTGGATA |

**For sequencing of a novel plasmid in S23A**

| **Contigs** | Forward Primer | Reverse Primer |
| --- | --- | --- |
| Contig64-Contig141 | ATCAGCGTTTTCAAGCCATC | ATGTGCGCCCCGCTGGTTCA |
| Contig141-Contig143 | CGTTTTGGTCTTGCCATTTC | AGAAACCGGCTTGATTGAGA |
| Contig143-Contig158 | CGATATTCGCGTTTAACCTCA | AGGATTACTTTCGCATTGTTC |
|  |  | GGAGGGCTTGACCTTGATA |
| Contig158-Contig504 | AGCTAAGCAGCACCCGAAA | TGTTTTGTTGCCAACGCT |
|  |  | AGTCACGACTCAACACATCA |
| Contig504-Contig142 | TGGCAACAAAaCAGAAAGG | CAAGCCGCTAAAGAACTACGA |
| Contig142-Contig457 | TTCCTCTCGCCTTAAATCTCA | AAAATGGGATTACGGCCAAG |
| Contig457-Contig595 | CGCATATTTCTTCGCTGCTT | GCTCAAAGCGGGTTTAGTC |
| Contig595-Contig91 | CGACCGCCTATAATGACGA | ACTTATTTagCGGCGGATGACAT |
| Contig91-Contig90 | TCGCCTTCAAGCAATTCAC | CGTAACGGTCAGATCCAA |
| Contig90-Contig59 | TTCTTCGCCTTGTTGGATCT | AACCACAACCAATGCCTACC |
| Contig59-Contig98 | AATCGGTTTCCGTTTTGTC | GCGGTGAAAAAGTTGTAGG |
| Contig98-Contig69 | tTTTCGCCCTCActGCTTTG | TGGTTGAAGGGTTCCGTATG |
| Contig69-Contig78 | CACGAATCAGaAATgGCAC | CAATACCAATCCAAGCTAAGCC |
| Contig78-Contig437 | ACTACCACCTTTGCCATTTAGAC | GGGCAAAAGAAGCAAAACAA |
| Contig437-Contig66 | CTGATTCCGAAACCATCCAC | GGCGGAAAATACATGCTGA |
| Contig66-Contig64 | CGTTTTTCGCCTCTGTCATC | GAGGAATTTAAGCGtTTTTGCtG |

**For PCR and sequencing confirmation of the integration of plasmid-related sequence in the genome of S23A and I23C**

| Forward Primer | Reverse Primer |
| --- | --- |
| TACTGGTTACAGGCAAATGGG | TTATCTGCGTTTGCTAGCGC |
| TCAATTAGCCTCTGTCTCGG | TCACATCATAAGAGAGGGTGC |
| TTTGCCATTAATGGCTCCG | AAATCCAGCCCGGTATCC |
| TTATCTGCGTTTGCTAGCGC | AAATCCAGCCCGGTATCC |
| AGTTCGGTGTAATGGTTTCGC | TGCCGTATTACCAGCTATTCC |
| AGAATGAAACGGCTTCACCG | TGCCGTATTACCAGCTATTCC |

**Gap closing of the SSP gene cluster in S23A**

| **Contigs** | Forward Primer | Reverse Primer |
| --- | --- | --- |
| Contig462-Contig52 | GCGAAGCCATCCTTATTGAA | GATGTTGCGGGTAAAGATCG |
| Contig52-Contig473 | CAATGCGCCAAATGAAAAAT | TCTATGGAGCATTTTAAATCCATTA |
| Contig473-Contig537 | TCAACAGAACCATCTTTAGAACCA | TGGGGAGTTTTCTTAACAATGTG |
| Contig537-Contig558 | AACATCCCATCCTGCAAAAA | TGGTAAGCATCTGCCATATGAA |
| Contig558-contig430 | TCCCAAGCTACATTTTGTGC | AGCACATCTTGGGAAAATGA |
| Contig430-Contig503 | ATGTGCCATCAGTTGAACCA | GATGGGGTCAACAAATCCAG |
| Contig503-Contig309 | GCAAGGCTTTGGGTAACAAG | AGTTGCCACACGCTAATTCC |

**Gap closing of the SSP gene cluster in I23C**

| **Contigs** | Forward Primer | Reverse Primer |
| --- | --- | --- |
| Contig31-Contig372 | CAACTATGGGTGCCGGAAG | CAGCAGACAATTTTGCCACA |
|  | AATTAGCAATGCGCCAAATG | TGTGTTGACAGATGAAGCTGAA |
| Contig372-Contig349 | TATCGCCAATAATCCGAACG | CCACTTTCTAACATTTCGGTCA |
|  | CCTGATCGACAGAGTGAGATACA | CAGAAATAATTAACGTTGAAAATGC |
| Contig349-Contig261 | TCTAAGAAAGAAAAATTGTATTGGTCA | TGCTTTTCGTGCATACAAGG |
|  | CATTGGTATTTGCAGATTCATCA | TCTAAGAAAGAAAAATTGTATTGGTCA |
| Contig261-Contig141 | TCCCAAGCTACATTTTGTGC | AAACTGTCCGCACGGTTATG |
|  | GGCTGGAAACCACAATATCAA | GGGATATGCCAAGCGTGAT |

**For verification of the inverted region in the SSP gene cluster in I23C**

| Forward Primer | Reverse Primer |
| --- | --- |
| ATGTGCCATCAGTTGAACCA | TCTGTAGGCGGTATATCAGCTTT |
| TCGGACCTTGAACAATTTCAT | GGACGCTATTTTATTCCGATCA |
| AACTGAGAATGCAACGGTTT | TGGACAACATATGCCATCAGA |
| AAAACGGCGATTCAAAATAGA | TCTGATGGCATATGTTGTCCA |
| GGTTTTAATACCCATTAACAATGC | AAAATTAAAGATGGTGTTGATATTGC |
| TGTATAAAACGGCGATTCAAAA | TTCAAACTGGAATGATGTACGC |
| GGGTGCATACGATGACAGAA | GGAGCTAACGTTATTGATCTTCC |
| AAAACGGCGATTCAAAATAGA | TCTGGTGGTGTTCAAACTGG |
