# Supplementary material for: Genomic Stability of Aggregatibacter actinomycetemcomitans during Persistent Oral Infection in Human
Source: PLoS One. 2013 Jun 18;8(6):e66472. doi: 10.1371/journal.pone.0066472 (PMC3688926; doi:10.1371/journal.pone.0066472)
Supplement: Table S4 — Cluster ID and product of the 150 core genes used for phylogenetic analysis of A. actinomycetemcomitans. (DOCX) [file pone.0066472.s008.docx]

**Supporting Information**

**Table S4.** Cluster ID and product of the 150 core genes used for phylogenetic analysis of *A. actinomycetemcomitans*.

| Cluster ID | Product Description | Expected Length | Cluster ID | Product Description | Expected Length |
| --- | --- | --- | --- | --- | --- |
| p-cluster00040 | ponB, mrcB | 2394 | p-cluster00599 | rpL2, rplB | 822 |
| p-cluster00057 | ycbY | 2163 | p-cluster00602 | truA, hisT | 819 |
| p-cluster00112 | msbA, ywjA | 1749 | p-cluster00619 | DeoR family | 798 |
| p-cluster00122 | fruA | 1698 | p-cluster00628 | comL | 795 |
| p-cluster00138 | cstA | 1599 | p-cluster00626 | atpB | 789 |
| p-cluster00162 | emrB | 1518 | p-cluster00627 | lpxA | 789 |
| p-cluster00169 | murC | 1494 | p-cluster00686 | surE | 786 |
| p-cluster00196 | pntB | 1485 | p-cluster00643 | COG3022 | 780 |
| p-cluster00187 | hyfD | 1446 | p-cluster00645 | vacJ | 777 |
| p-cluster00236 | folC | 1392 | p-cluster00646 | hycG, hevG | 777 |
| p-cluster00235 | Na+/H+ antiporter NhaC | 1386 | p-cluster00658 | ydfG | 768 |
| p-cluster00220 | atpD | 1374 | p-cluster00664 | rph | 762 |
| p-cluster00225 | hemN | 1368 | p-cluster00668 | udp, mtaP, deoD | 759 |
| p-cluster00241 | mrsA | 1338 | p-cluster00673 | 23S rRNA pseudouridine synthase D | 753 |
| p-cluster00246 | srmB, deaD, rhlB | 1332 | p-cluster00676 | trmD | 753 |
| p-cluster00261 | murD | 1317 | p-cluster00685 | act | 741 |
| p-cluster00258 | eno | 1311 | p-cluster00697 | fabG, envM | 729 |
| p-cluster00265 | pepP | 1302 | p-cluster00702 | yhbG | 726 |
| p-cluster00269 | lamB | 1302 | p-cluster00707 | putative M22 peptidase-like protein YeaZ | 723 |
| p-cluster00272 | kdtA, waaA | 1284 | p-cluster00715 | rpS3, rpsC | 708 |
| p-cluster00279 | hemY | 1278 | p-cluster00725 | ribonuclease T2 family | 696 |
| p-cluster00280 | thrC\ | 1275 | p-cluster00727 | pfs, mtn | 693 |
| p-cluster00330 | galK | 1248 | p-cluster00730 | rpl1, rplA | 690 |
| p-cluster00292 | nqrF | 1236 | p-cluster00774 | yrbC | 690 |
| p-cluster00324 | sucC | 1224 | p-cluster00749 | ATP-binding protein | 684 |
| p-cluster00299 | fabB | 1221 | p-cluster00744 | metI | 678 |
| p-cluster00317 | lpxB | 1185 | p-cluster00748 | dsbA | 675 |
| p-cluster00336 | integral membrane protein | 1140 | p-cluster00758 | minC | 675 |
| p-cluster00342 | anhydro-N-acetylmuramic acid kinase | 1131 | p-cluster00756 | ccmB | 666 |
| p-cluster00346 | nagZ, hexA | 1128 | p-cluster00775 | hyfE | 639 |
| p-cluster00363 | ychF, obg | 1128 | p-cluster00813 | slyD | 639 |
| p-cluster00347 | ribD | 1125 | p-cluster00781 | tmk | 633 |
| p-cluster00349 | rodA, mrdB | 1122 | p-cluster00785 | nqrD | 630 |
| p-cluster00353 | asd | 1113 | p-cluster00789 | pyrR, upp | 627 |
| p-cluster00356 | gpcE, ispG | 1104 | p-cluster00792 | rhtC | 624 |
| p-cluster00412 | purM | 1095 | p-cluster00809 | protein YcfC | 612 |
| p-cluster00367 | apbE | 1089 | p-cluster00826 | putative glycerol-3-phosphate acyltransferase PlsY | 606 |
| p-cluster00368 | aroB | 1089 | p-cluster00818 | rpL4, rplD | 603 |
| p-cluster03251 | nrfF | 1086 | p-cluster00819 | recR | 603 |
| p-cluster00378 | aroC | 1074 | p-cluster00822 | hyfA | 600 |
| p-cluster00381 | conserved permease | 1068 | p-cluster00827 | D-fructose-6-phosphate amidotransferase | 594 |
| p-cluster00383 | murG | 1065 | p-cluster00829 | nudE | 591 |
| p-cluster00428 | bioB | 1059 | p-cluster00836 | dcd | 588 |
| p-cluster00415 | lpxD, firA | 1038 | p-cluster00832 | mob | 585 |
| p-cluster00431 | ydeZ | 1011 | p-cluster00847 | ruvC | 573 |
| p-cluster00430 | trpS | 1005 | p-cluster00856 | protein in HemN 3'region | 561 |
| p-cluster00451 | thiL | 996 | p-cluster00857 | integral membrane protein | 561 |
| p-cluster00456 | rluD, sfhB | 996 | p-cluster00860 | yeaY, slp, rnd | 558 |
| p-cluster00442 | pheS | 990 | p-cluster00867 | comB | 558 |
| p-cluster00443 | rpoA | 990 | p-cluster00866 | ccmG, dsbE | 552 |
| p-cluster00446 | trpX, miaA | 987 | p-cluster00876 | rpL6, rplF | 534 |
| p-cluster00452 | oppD | 984 | p-cluster00878 | hslV, clpQ | 531 |
| p-cluster00458 | cysB, cbl, gltC | 972 | p-cluster00879 | ppa, ipyR | 531 |
| p-cluster00461 | ydeW | 966 | p-cluster00885 | actF, fldA | 525 |
| p-cluster00462 | pfkA | 966 | p-cluster00887 | periplasmic protein | 522 |
| p-cluster00465 | fatD, yclN, ceuB | 966 | p-cluster00911 | YfbU family protein | 507 |
| p-cluster00479 | yfcB | 951 | p-cluster00903 | tpx | 501 |
| p-cluster00491 | fabD | 948 | p-cluster00907 | rps5, rpsE | 501 |
| p-cluster00484 | tkt | 945 | p-cluster00908 | pgpA | 501 |
| p-cluster00487 | fruk, lacC | 942 | p-cluster00912 | kdtB, coaD | 495 |
| p-cluster00493 | ftsX | 936 | p-cluster06280 | COG1238 | 489 |
| p-cluster00500 | rfaD | 927 | p-cluster00926 | ribH | 474 |
| p-cluster00502 | HflC | 927 | p-cluster00927 | YccF | 474 |
| p-cluster00549 | hslO, hsp33 | 909 | p-cluster00928 | IscR | 474 |
| p-cluster00517 | mepA | 903 | p-cluster00932 | COG1607 | 471 |
| p-cluster00569 | menB | 897 | p-cluster00968 | atpC | 456 |
| p-cluster00540 | sapC | 888 | p-cluster00955 | holD | 453 |
| p-cluster00541 | D-3-phosphoglycerate dehydrogenase | 888 | p-cluster00975 | excinuclease ATPase subunit | 447 |
| p-cluster00542 | nagC, glcK | 888 | p-cluster00962 | thioester dehydrase family | 444 |
| p-cluster00555 | sucD | 879 | p-cluster00981 | integral membrane protein | 441 |
| p-cluster00550 | citE, cilB | 876 | p-cluster00966 | organic solvent tolerance protein | 435 |
| p-cluster00556 | N-acetylmannosamine kinase |  | p-cluster00976 | COG2050 | 432 |
| p-cluster00562 | ksgA | 864 | p-cluster00982 | hslR | 417 |
| p-cluster00577 | AfeD, yfeD | 849 | p-cluster00984 | smpA | 414 |
| p-cluster00597 | icc | 825 | p-cluster00988 | excinuclease ABC subunit A | 414 |
|  |  |  |  |  |  |
