# Supplementary material for: Genomic Stability of Aggregatibacter actinomycetemcomitans during Persistent Oral Infection in Human
Source: PLoS One. 2013 Jun 18;8(6):e66472. doi: 10.1371/journal.pone.0066472 (PMC3688926; doi:10.1371/journal.pone.0066472)
Supplement: Table S5 — PCR analysis of genes of disparity of the paired strains. (DOCX) [file pone.0066472.s009.docx]

**Supporting Information**

**Table S5.** PCR analysis of genes of disparity of the paired strains

| **p-cluster** | **Length (bp)** | **Product Description** | **Predicted Gene Presence/Absence in the Paired Strains S23A/I23C** | **Results by PCR analysis** |
| --- | --- | --- | --- | --- |
| 02269 | 474 | Lytic transglycosylase catalytic | +/-^a^ | **+/-** |
| 02280 | 576 | Site-specific recombinase | +/-^a^ | **+/-** |
| 02561 | 1119 | Nickase | +/-^a^ | **+/-** |
| 02578 | 428 | Replication protein | +/-^a^ | **+/-** |
| 02790 | 282 | Hypothetical protein | +/-^a^ | **+/-** |
| 03521 | 711 | Hypothetical protein | +/-^a^ | **+/-** |
| 03622 | 615 | Hypothetical protein | +/-^a^ | **+/-** |
| 03948 | 387 | Hypothetical protein | +/-^a^ | **+/-** |
| 15527 | 195 | Hypothetical protein | +/-^b^ | **+/-** |
| 01320 | 126 | Hypothetical Protein | -/+ | +/+ |
| 01668 | 120 | Hypothetical Protein | -/+ | +/+ |
| 01699 | 150 | Hypothetical Protein | -/+ | +/+ |
| 01967 | 189 | Hypothetical protein | -/+ | +/+ |

| **p-Cluster** | **Length (bp)** | **Product Description** | **Predicted Gene Presence/Absence in the Paired Strains SCC393/A160** | **Results by PCR analysis** |
| --- | --- | --- | --- | --- |
| 13483 | 189 | Hypothetical protein | +/- | +/+ |
| 02391 | 174 | Hypothetical protein | -/+ | +/+ |
| 01053 | 282 | COG3668:Plasmid stabilization system | +/- | +/+ |
| 01138 | 123 | Hypothetical Protein | +/- | +/+ |
| 01300 | 126 | Hypothetical Protein | +/- | +/+ |
| 01480 | 153 | Sucrose-6-phosphate hydrolase | +/- | +/+ |
| 04441 | 120 | hypothetical protein | +/- | +/+ |
| 01506 | 489 | Sulfatase | +/- | +/+ |
| 01753 | 153 | Hypothetical Protein | +/- | +/+ |
| 02140 | 114 | Hypothetical Protein | +/- | +/+ |
| 02923 | 156 | Hypothetical Protein | +/- | +/+ |
| 01552 | 528 | tfoX: DNA transformation protein | +/- | +/+ |
| 01556 | 279 | tadF: tight adherence protein F | +/- | +/+ |
| 01651 | 144 | IS1016 transposase | +/- | +/+ |
| 13977 | 201 | Hypothetical Protein | +/- | +/+ |
| 01751 | 153 | Hypothetical Protein | +/- | +/+ |
| 01876 | 168 | Hypothetical Protein | +/- | +/+ |
| 01894 | 174 | Hypothetical Protein | +/- | +/+ |
| 01967 | 189 | Hypothetical Protein | +/- | +/+ |
| 02004 | 654 | hypothetical protein | +/- | +/+ |
| 02062 | 234 | protein HipA | +/- | +/+ |
| 02030 | 366 | COG0614: ABC-type Fe3+-hydroxamate | +/- | +/+ |
| 02061 | 126 | hypothetical protein | +/- | +/+ |
| 02110 | 234 | Hypothetical Protein | +/- | +/+ |
| 02134 | 126 | Hypothetical Protein | +/- | +/+ |
| 02455 | 120 | Hypothetical Protein | +/- | +/+ |
| 02841 | 150 | Hypothetical Protein | +/- | +/+ |
| 04804 | 243 | Hypothetical Protein | +/- | +/+ |
| 12497 | 156 | hypothetical protein | +/- | +/+ |
| 12703 | 270 | hypothetical protein | +/- | +/+ |
| 12864 | 123 | Hypothetical Protein | +/- | +/+ |
| 13511 | 114 | Hypothetical Protein | +/- | +/+ |
| 14115 | 237 | Hypothetical Protein | +/- | +/+ |

| **p-cluster** | **Length (bp)** | **Product Description** | **Predicted Gene Presence/Absence in the Paired Strains SCC1398/SCC4092** | **Results by PCR analysis** |
| --- | --- | --- | --- | --- |
| 01799 | 292 | Cell filamentation protein Fic-related protein | +/- | +/+ |
| 01550 | 117 | Hypothetical Protein | +/- | +/+ |
| 00457 | 114 | Hypothetical Protein | -/+ | +/+ |

| **p-cluster** | **Length (bp)** | **Product Description** | **Predicted Gene Presence/Absence in the Paired Strains SCC2302/AAS4a** | **Results by PCR analysis** |
| --- | --- | --- | --- | --- |
| 12007 | 147 | Hypothetical protein | +/- | +/+ |
| 03035 | 259 | Bacteriophage Mu GP27-like protein | -/+^a^ | +/+ |
| 12011 | 117 | Hypothetical protein | +/- | Undecided^c^ |
| 12012 | 120 | Hypothetical protein | +/- | Undecided^c^ |

^a^ Genes identified by the concordant results of WGS and CGH.

^b^ Identified by its position next to a confirmed gene of disparity in strains S23A/I23C.

^c^ Multiple bands of amplicons were detected in repeated PCR analysis with different sets of primers.
